# Mediterranean diet adherence, healthy sleep, and lung cancer risk: a prospective study of 192,034 UK Biobank participants

**DOI:** 10.1007/s00394-026-04080-x

**Published:** 2026-08-27

**Authors:** Yiming Chen, Enyu Tong, Xiaoman Jin, Yufeng Rao, Evan Y. W. Yu, Abdulmohsen H. Al-Zalabani, Maurice Zeegers, Anke Wesselius

**Affiliations:** 1 School of Nutrition and Translational Research in Metabolism, Maastricht University, Maastricht, Limburg the Netherlands; 2 Department of Epidemiology, Maastricht University, Maastricht, Limburg the Netherlands; 3 Department of Health, Ethics and Society, Maastricht University, Maastricht, Limburg the Netherlands; 4 Key Laboratory of Environmental Medicine and Engineering of Ministry of Education, and Department of Epidemiology & Biostatistics, School of Public Health, Southeast University, Nanjing, Jiangsu China; 5 Department of Family and Community Medicine, College of Medicine, Taibah University, Madinah, Saudi Arabia

**Keywords:** Lung cancer, Mediterranean diet, Sleep, Lifestyle factors, UK Biobank

## Abstract

**Purpose:**

Diet and sleep have each been associated with cancer risk, but their joint association with lung cancer risk remains unclear. We investigated the association between a joint Mediterranean diet-sleep pattern and lung cancer risk in the UK Biobank. We also evaluated whether siesta modified the association between Mediterranean diet adherence and lung cancer risk.

**Methods:**

Participants were drawn from the UK Biobank. Mediterranean diet adherence was assessed using an adapted Mediterranean Diet Adherence Screener, and sleep was evaluated using a composite score based on five sleep behaviors. Mediterranean diet and sleep scores were categorized into tertiles and jointly classified into nine levels of a diet-sleep pattern. Associations were examined using Fine-Gray proportional subdistribution hazards models that accounted for competing risks from all-cause mortality, yielding subdistribution hazard ratios (SHRs) and 95% confidence intervals (CIs).

**Results:**

A total of 192,034 participants (median age 57 years; 54% were female) were included and followed for a median of 14 years, during which 1156 incident lung cancer cases occurred. Compared with participants in the lowest level, those in the highest level had a 43% lower risk of lung cancer (SHR 0.57, 95% CI 0.41–0.79; *P* = 0.001). No evidence was found that siesta modified the association between Mediterranean diet adherence and lung cancer risk.

**Conclusions:**

The joint Mediterranean diet-sleep pattern was associated with a lower risk of lung cancer, although this finding should be interpreted as an observational association rather than evidence of causality.

**Supplementary Information:**

The online version contains supplementary material available at https://doi.org/10.1007/s00394-026-04080-x.

## Introduction

Cancer is a major global public health challenge. According to a report from the World Health Organization, approximately 10 million individuals died of cancer in 2020, accounting for one-sixth of all deaths globally [1]. Among all cancer types, lung cancer is the most commonly diagnosed and the leading cause of cancer mortality worldwide [1, 2]. Depending on the specific stage and region, the five-year survival rate for lung cancer ranges from 4 to 17% [3]. These observations underscore the importance of identifying modifiable factors relevant to lung cancer prevention.

In addition to advances in cancer treatment, primary prevention through lifestyle modification is widely recognized as a key strategy for reducing the cancer burden. Lifestyle factors, particularly sleep and diet, play crucial roles in overall health. Unhealthy sleep and diet share adverse mechanisms such as systemic inflammation and reduced antioxidant capacity [4, 5]. Unhealthy sleep patterns, such as insomnia, short sleep duration, and daytime dozing, have been reported to be associated with increased risks of cardiovascular disease, type 2 diabetes, and cancer [6–8]. Similarly, unhealthy dietary patterns, such as low consumption of vegetables, legumes, and fruits and higher intake of red meat and processed foods, are associated with comparable adverse outcomes [9, 10]. These observations underscore the importance of considering the two factors jointly as a diet-sleep pattern in cancer prevention.

However, existing epidemiological studies have mainly examined the associations of diet or sleep with disease risk independently when assessing cancer risk [11–13]. Quantitative research that integrates both dietary and sleep behaviors into a joint diet-sleep pattern and systematically evaluates their association with cancer risk remains limited. Furthermore, existing studies assessing lifestyle and cancer risk have mainly used Cox regression models without accounting for competing risks from all-cause mortality [14–17].

Therefore, we primarily examined the association between a joint Mediterranean diet-sleep pattern and lung cancer risk using Fine-Gray proportional subdistribution hazards models that accounted for competing mortality. The dietary component was based on the Mediterranean diet, a widely recognized healthy dietary pattern characterized by high consumption of plant-based foods, olive oil, and fish, and limited intake of red meat and processed foods [13, 18, 19]. Furthermore, because the Mediterranean diet is often embedded in a broader cultural context that includes siesta, a habit that has sometimes been associated with adverse health outcomes [20, 21], we also explored whether siesta modified the association between Mediterranean diet adherence and lung cancer risk. Exploratory analyses were conducted for several other common cancers to assess whether any observed association was specific to lung cancer.

## Materials and methods

### Source of data and study population

This study used data from the UK Biobank, a large-scale cohort including more than 500,000 individuals aged 37 to 73 years from England, Scotland, and Wales between 2006 and 2010 [22]. Every individual provided digital consent and engaged in a self-completed touch-screen questionnaire, a short computer-based interview, as well as physical and functional evaluations. Additionally, biological specimens such as blood, urine, and saliva were gathered for laboratory analysis. Participants were followed from recruitment through September 2023 until incident lung cancer, death, loss to follow-up, or the end of follow-up, whichever came first.

Dietary intake data in the UK Biobank were collected using the 24-h dietary recall questionnaire (Oxford WebQ) [23]. Because only 70,650 participants completed the Oxford WebQ at the initial dietary assessment (April 2009 to September 2010), we included all participants who completed the questionnaire at least once, either at the initial dietary assessment or during one of the four follow-up assessment rounds, to maximize the sample size. Participants could complete the Oxford WebQ once during the initial dietary assessment and up to four additional times during the follow-up rounds (round 1: February to April 2011; round 2: June to September 2011; round 3: October to December 2011; round 4: April to June 2012). These recalls were not collected on consecutive days. Questionnaires were issued on different days of the week to capture variation between weekday and weekend dietary intake, and repeated administrations were intended to account for seasonal variation and provide a more stable estimate of habitual intake. Because this study focused on cancer incidence, participants who had been diagnosed with cancer at baseline were excluded.

Ethical approval was granted by the Northwest Multi-Centre Research Ethics Committee. All participants gave written informed consent before enrollment in the study, which was conducted in accordance with the principles of the Declaration of Helsinki.

### Selection of variables

All the included variables can be broadly categorized into four categories (**S**upplementary Table S1):

- Dietary consumption variables: according to the adapted version of MEDAS detailed below, the following 11 predefined broad categories of food should be considered: olive oil, vegetables, fruits, red meat, sweetened or carbonated drinks, wine, legumes, fish/shellfish, sweets/pastries, nuts, and white meat. Ultimately, 107 food variables were eligible and were included. Ultra-processed foods were not classified or analyzed as a separate dietary exposure in this study.
- Sleep-related factors: (1) sleep duration: participants reported their average sleep duration in hours over a 24-h period; (2) insomnia: frequency of trouble falling asleep or waking up in the middle of the night was categorized as “never/rarely,” “sometimes,” and “usually;” (3) snoring: assessed with a yes/no response to whether a partner, relative, or friend had complained about it; (4) daytime dozing: likelihood of dozing off during the day was rated on a four-point scale: “never/rarely,” “sometimes,” “often,” and “all of the time;” (5) chronotype: participants self-identified their chronotype as “definitely a ‘morning’ person,” “more a ‘morning’ than ‘evening’ person,” “more an ‘evening’ than a ‘morning’ person,” or “definitely an ‘evening’ person;” and (6) siesta: frequency of siesta was categorized as “never/rarely,” “sometimes,” and “usually.”
- Cancer incidence status defined by the 10th revision of the International Classification of Diseases codes (lung cancer: C34) and date-related variables.
- Covariates: (1) age at recruitment; (2) sex; (3) coffee consumption: defined by “How many cups of coffee do you drink each day?”; (4) smoking status: an existing derived variable from the UK Biobank database, categorized as “never,” “previous,” and “current.” This variable was derived from “current tobacco smoking” and “past tobacco smoking,” with “never” indicating participants who had never smoked tobacco, “previous” indicating former smokers, and “current” indicating current smokers; (5) alcohol status: an existing derived variable from the UK Biobank database, categorized as “never,” “previous,” and “current.” This variable was derived from “alcohol intake frequency” and “former alcohol drinker status,” with “never” indicating participants who had never consumed alcohol, “previous” indicating former drinkers, and “current” indicating current drinkers; (6) activity intensity: measured with International Physical Activity Questionnaire and categorized into “low,” “moderate,” and “high;” (7) household income: defined by “What is the average total income before tax received by your HOUSEHOLD? (£)” and categorized into “less than 18,000,” “18,000 to 30,999,” “31,000 to 51,999,” “52,000 to 100,000,” and “greater than 100,000;” (8) employment status: categorized into “in paid employment or self-employed,” “retired,” and “other status,” and (9) Body Mass Index (BMI): based on the physical measurement data at recruitment and calculated with the formula that *BMI* = *weight*(kg)/*height*(m)^*2*.

These covariates were selected a priori based on their established or potential role as confounders in the association between Mediterranean diet adherence, healthy sleep, and cancer risk. For categorical variables, response categories were used as provided in the UK Biobank touchscreen questionnaire for primary variables and in the UK Biobank database for derived variables, rather than being redefined by the authors.

### Assessment of Mediterranean diet

Mediterranean diet adherence was assessed using the Mediterranean Diet Adherence Screener (MEDAS) [24, 25]. As the Oxford WebQ does not capture all food components specified in the original MEDAS (e.g., it does not assess the daily consumption of olive oil in tablespoons), an adapted version of MEDAS using the 11 available broad food categories was utilized [26]. The original version of the MEDAS and the modified version with scoring criteria are detailed in Supplementary Tables S2 and S3.

For each completed 24-h dietary assessment, a MEDAS score was calculated. Among the included participants, 5760 had data available at all five time points (the initial assessment and rounds 1 to 4), while 76,523 had data available at only one time point. To better represent habitual dietary intake and mitigate the limitations of single-day recalls, we calculated the mean MEDAS score for each participant by averaging the scores across all available assessments. A higher mean MEDAS score indicates a greater degree of adherence to the Mediterranean diet.

### Assessment of sleep

A sleep score was constructed from five components: sleep duration, insomnia, snoring, daytime dozing, and chronotype. Each component was considered low risk (score of 1) according to the following criteria [6]: (1) sleep duration: seven to eight hours per day; (2) insomnia: “never/rarely”; (3) snoring: “no”; (4) daytime dozing: “never/rarely” or “sometimes”; (5) chronotype: “definitely a ‘morning’ person” or “more a ‘morning’ than ‘evening’ person”. Otherwise, a score of 0 was assigned. The total sleep score was calculated by summing the five binary components, with a higher score indicating healthier sleep.

### Statistical analysis

All statistical analyses were performed using R 4.4.0 and RStudio 2024.04.1 + 748. Descriptive statistics were presented as medians for continuous variables and frequencies (percentages) for categorical variables, stratified by sleep score tertiles based on sample quantiles.

After participant exclusion, the missing data proportion for each variable was below 20%. These proportions were as follows: coffee consumption (0.12%), smoking status (0.27%), alcohol status (0.10%), activity intensity (17.44%), household income (10.28%), employment status (0.28%), BMI (0.28%), olive oil item (9.22%), sleep duration (0.34%), insomnia (0.11%), snoring (6.63%), daytime dozing (0.28%), chronotype (10.42%), and siesta (0.09%). To preserve statistical power and reduce potential bias from complete-case analysis, missing data were handled using multiple imputation by chained equations implemented in the mice package in R. All variables included in the analysis were entered into the imputation model.

To account for competing risks from all-cause mortality during the follow-up period, a competing risk model was implemented using the Fine-Gray proportional subdistribution hazards model. Each analysis was initially adjusted for age and sex (model 1), and further adjusted for coffee consumption, smoking status, alcohol status, activity intensity, household income, employment status, and BMI (model 2). The independent associations between MEDAS/sleep score and lung cancer risk were first examined. In the main analyses, MEDAS and sleep scores were categorized into tertiles (T1-T3) and cross-classified to form nine levels of a joint diet-sleep pattern (level 1: T1 MEDAS and T1 sleep; level 2: T1 MEDAS and T2 sleep; level 3: T1 MEDAS and T3 sleep; level 4: T2 MEDAS and T1 sleep; …; and level 9: T3 MEDAS and T3 sleep). Level 1 of the joint diet-sleep pattern was used as the reference category. Subdistribution hazard ratios (SHRs) and their 95% confidence intervals (CIs) were calculated.

Analyses for both additive and multiplicative interactions were performed to assess any synergistic or antagonistic effects after dividing diet and sleep at their sample medians into upper (≥ 50th percentile) and lower (< 50th percentile) halves. In detail, additive interaction was measured with the Relative Excess Risk due to Interaction (RERI), the Attributable Proportion due to Interaction (AP), and the Synergy Index (S); multiplicative interaction was evaluated by comparing models with and without the interaction term between MEDAS and sleep scores using the likelihood-ratio test.

To assess the robustness of our findings, we conducted sensitivity analyses in which we simultaneously excluded participants with a follow-up period of less than two years and divided both the MEDAS and sleep scores at their sample medians, thereby creating a four-level joint diet-sleep pattern.

Furthermore, due to known differences in lung cancer incidence by sex [2], stratified analyses by sex were performed.

Given that smoking is a major risk factor for lung cancer, its potential effect-modifying role for the association between the joint diet-sleep pattern and lung cancer risk was investigated using the likelihood-ratio test. The potential effect-modifying role of siesta in the association between the Mediterranean diet and lung cancer was also investigated using the likelihood-ratio test.

A *P*-value of less than 0.05 was considered statistically significant.

## Results

### Characteristics of participants

**Fig. 1:**
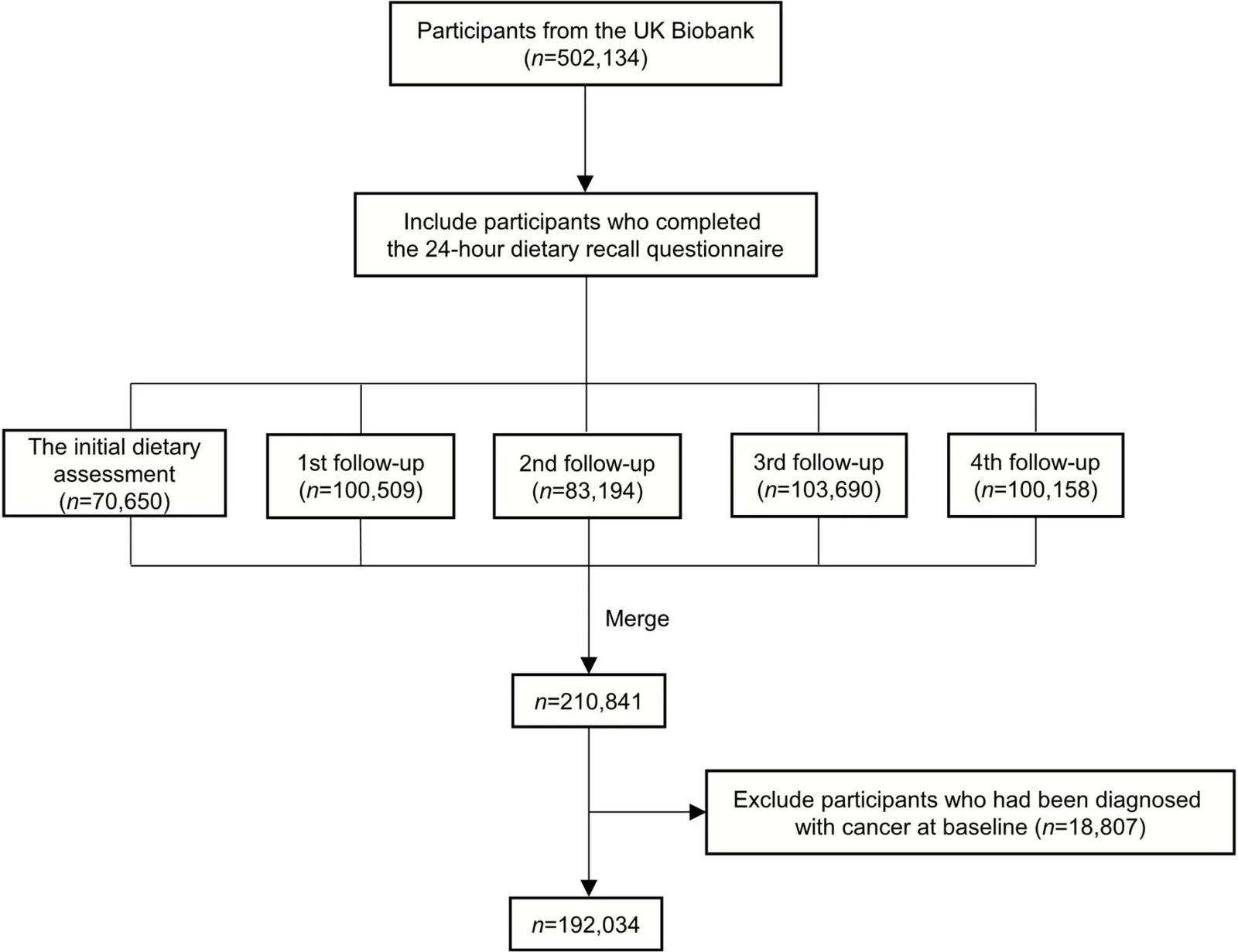
Exclusion flowchart

A total of 192,034 participants with a median follow-up duration of 14 years were included in this study. The exclusion flowchart is presented in Fig. 1. Among the included participants, the median age was 57 years, and 54.08% were female (Table 1). Although several participant characteristics varied across sleep score tertiles, the overall characteristics of participants were broadly similar.

**Table 1:** Characteristics of participants

Variables	Total (*n* = 192,034)	Sleep score		
		Tertile 1 (*n* = 70,741)	Tertile 2 (*n* = 78,424)	Tertile 3 (*n* = 42,869)
MEDAS score, median	3.33	3.00	3.33	3.50
Siesta,* n* (%)				
Never/rarely	114,383 (59.56)	39,632 (56.02)	47,488 (60.55)	27,263 (63.60)
Sometimes	68,958 (35.91)	26,919 (38.05)	27,818 (35.47)	14,221 (33.17)
Usually	8693 (4.53)	4190 (5.92)	3118 (3.98)	1385 (3.23)
Sex,* n* (%)				
Female	103,855 (54.08)	35,685 (50.44)	41,954 (53.50)	26,216 (61.15)
Male	88,179 (45.92)	35,056 (49.56)	36,470 (46.50)	16,653 (38.85)
Age, median	57	57	57	56
Body Mass Index, median	26.27	27.04	26.12	25.36
Coffee intake (cups/day), median	2	2	2	1
Alcohol status,* n* (%)				
Never	6285 (3.27)	2159 (3.05)	2560 (3.26)	1566 (3.65)
Previous	5840 (3.04)	2271 (3.21)	2317 (2.95)	1252 (2.92)
Current	179,909 (93.69)	66,311 (93.74)	73,547 (93.78)	40,051 (93.43)
Smoking status,* n* (%)				
Never	109,298 (56.92)	37,020 (52.33)	45,390 (57.88)	26,888 (62.72)
Previous	67,523 (35.16)	26,727 (37.78)	27,152 (34.62)	13,644 (31.83)
Current	15,213 (7.92)	6994 (9.89)	5882 (7.50)	2337 (5.45)
Activity intensity,* n* (%)				
Low	34,827 (18.14)	14,789 (20.91)	13,687 (17.45)	6351 (14.81)
Moderate	81,210 (42.29)	29,971 (42.37)	33,191 (42.32)	18,048 (42.10)
High	75,997 (39.57)	25,981 (36.73)	31,546 (40.22)	18,470 (43.08)
Household income,* n* (%)				
Less than 18,000	30,311 (15.78)	11,992 (16.95)	12,159 (15.50)	6160 (14.37)
18,000 to 30,999	46,792 (24.37)	17,574 (24.84)	18,893 (24.09)	10,325 (24.09)
31,000 to 51,999	54,430 (28.34)	19,941 (28.19)	22,377 (28.53)	12,112 (28.25)
52,000 to 100,000	46,597 (24.26)	16,576 (23.43)	19,225 (24.51)	10,796 (25.18)
Greater than 100,000	13,904 (7.24)	4658 (6.58)	5770 (7.36)	3476 (8.11)
Employment status,* n* (%)				
Employed	109,677 (57.11)	39,674 (56.08)	45,171 (57.60)	24,832 (57.93)
Retired	52,540 (27.36)	19,560 (27.65)	21,406 (27.30)	11,574 (27.00)
Other status	29,817 (15.53)	11,507 (16.27)	11,847 (15.11)	6463 (15.08)

### Primary results

Among the participants, 1156 were newly diagnosed with lung cancer during the follow-up.

Both higher Mediterranean diet adherence and healthier sleep were independently associated with a reduced risk of lung cancer after full adjustment. For the MEDAS score, participants in the highest tertile (T3) had a 21% lower risk (SHR 0.79, 95% CI 0.69–0.92, *P* = 0.002) compared to the lowest (T1). Similarly, those in the highest sleep score tertile (T3) had a 26% lower risk (SHR 0.74, 95% CI 0.62–0.88, *P* = 0.001) (Supplementary Tables S4 and S5).

In the main analysis, a graded inverse association was observed between the joint diet-sleep pattern and lung cancer risk (Table 2). Most notably, participants in the highest level exhibited a 43% lower risk of lung cancer (SHR 0.57, 95% CI 0.41–0.79, *P* = 0.001) compared to those at the lowest. Analyses for both additive (RERI (95% CI): 0.09 (− 0.14–0.32); AP (95% CI): 0.14 (− 0.21–0.50); S (95% CI): 0.81 (0.47–1.56)) and multiplicative (χ^2^ = 0.12, *P* = 0.727) interactions indicated no synergistic or antagonistic effect. No statistically significant interaction between smoking status and the joint diet-sleep pattern was observed (χ^2^ = 6.73, *P* = 0.978).

**Table 2:** The association between the joint diet-sleep pattern and lung cancer risk

Joint diet-sleep pattern	Model 1^a^		Model 2^b^	
	SHR (95% CI)	*P*-value	SHR (95% CI)	*P*-value
Level 1	1		1	
Level 2	**0.75 (0.60–0.94)**	**0.013**	0.84 (0.67–1.04)	0.110
Level 3	**0.75 (0.61–0.94)**	**0.012**	0.85 (0.69–1.06)	0.160
Level 4	0.91 (0.77–1.08)	0.260	1.02 (0.86–1.21)	0.800
Level 5	**0.66 (0.53–0.83)**	** < 0.001**	**0.80 (0.64–1.00)**	**0.047**
Level 6	**0.62 (0.50–0.77)**	** < 0.001**	**0.78 (0.62–0.97)**	**0.025**
Level 7	**0.61 (0.48–0.77)**	** < 0.001**	**0.76 (0.59–0.96)**	**0.023**
Level 8	**0.51 (0.38–0.71)**	** < 0.001**	**0.66 (0.48–0.91)**	**0.011**
Level 9	**0.42 (0.30–0.58)**	** < 0.001**	**0.57 (0.41–0.79)**	**0.001**

*SHR* subdistribution hazard ratio, *CI* confidence interval

^a^Adjusted for age and sex

^b^Further adjusted for all covariates

Exploratory analyses for other common cancers did not show similarly consistent associations (Supplementary Table S6).

### Sensitivity analyses

After excluding participants with a follow-up period of less than two years and dividing the MEDAS and sleep scores into upper and lower halves, the sensitivity analysis confirmed the robustness of the primary findings, with the inverse association remaining strong and statistically significant (Table 3).

**Table 3:** Sensitivity analysis for the joint diet-sleep pattern and lung cancer risk after full adjustment

Joint diet-sleep pattern^a^	Events/total^b^	SHR (95% CI)	*P*-value
Level 1	540/78,804	1	
Level 2	375/70,109	**0.78 (0.68–0.89)**	** < 0.001**
Level 3	87/20,788	**0.74 (0.59–0.92)**	**0.008**
Level 4	77/22,024	**0.60 (0.47–0.77)**	** < 0.001**

*SHR* subdistribution hazard ratio, *CI* confidence interval

^a^Created by dichotomizing both the MEDAS and sleep scores at their sample medians and cross-classifying them

^b^Participants with follow-up less than 2 years were excluded

### Stratified analyses by sex

Among females, the following levels of the joint diet-sleep pattern were associated with a lower risk: level 8 (SHR 0.61, 95% CI 0.40–0.94, *P* = 0.025), and level 9 (SHR 0.49, 95% CI 0.32–0.76, *P* = 0.001). Among males, the following levels of the joint diet-sleep pattern were associated with a lower risk: level 6 (SHR 0.67, 95% CI 0.47–0.96, *P* = 0.027), and level 7 (SHR 0.70, 95% CI 0.49–0.99, *P* = 0.046) (Table 4).

**Table 4:** Stratified analysis by sex after full adjustment

Joint diet-sleep pattern	Female (*n* = 103,855)			Male (*n* = 88,179)		
	Events/total	SHR (95% CI)	*P*-value	Events/total	SHR (95% CI)	*P*-value
Level 1	121/16,325	1		157/19,817	1	
Level 2	58/9043	0.89 (0.65–1.22)	0.470	51/8259	0.78 (0.57–1.07)	0.130
Level 3	69/10,317	0.89 (0.66–1.20)	0.440	45/6980	0.81 (0.58–1.13)	0.210
Level 4	102/18,130	0.90 (0.69–1.17)	0.420	160/19,639	1.12 (0.90–1.39)	0.320
Level 5	58/10,856	0.83 (0.61–1.13)	0.240	48/8838	0.76 (0.55–1.05)	0.093
Level 6	72/12,968	0.85 (0.63–1.14)	0.270	39/7993	**0.67 (0.47–0.96)**	**0.027**
Level 7	48/10,621	0.80 (0.58–1.12)	0.200	40/8636	**0.70 (0.49–0.99)**	**0.046**
Level 8	25/7023	**0.61 (0.40–0.94)**	**0.025**	20/4169	0.73 (0.46–1.16)	0.180
Level 9	25/8572	**0.49 (0.32–0.76)**	**0.001**	18/3848	0.71 (0.44–1.16)	0.170

*SHR* subdistribution hazard ratio, *CI* confidence interval

### The effect-modifying role of siesta

The analysis provided no evidence that siesta modified the association between MEDAS scores and lung cancer risk (χ^2^ = 3.09, *P* = 0.079).

## Discussion

The principal finding of this large prospective study is that the joint Mediterranean diet-sleep pattern was associated with lower lung cancer risk. In contrast, no similarly consistent associations were observed in exploratory analyses of other common cancers. Given the observational design, this finding should be interpreted cautiously and does not establish a causal effect.

### Inverse association between the joint diet-sleep pattern and lung cancer

Our results confirm the independent inverse association of the Mediterranean diet with lung cancer, aligning with previous meta-analyses. For instance, a 2022 meta-analysis by Du et al. [27] reported a risk reduction for lung cancer among individuals with the highest adherence to the Mediterranean diet, a finding our large prospective study robustly supports.

More novel is our observation regarding sleep. While previous studies have often linked poor sleep to increased mortality or higher cancer risk [5, 8], few have focused specifically on lung cancer with a multidimensional sleep score. Our finding that healthier sleep is associated with a lower lung cancer risk, even after extensive adjustment for smoking, adds a new layer to the understanding of sleep as a potential modifiable risk factor for this specific malignancy.

The most important finding was that the joint diet-sleep pattern combining Mediterranean diet adherence and healthy sleep showed a clear gradient association with lung cancer risk. Individuals in the highest level of the joint diet-sleep pattern had a 43% lower risk compared to those in the lowest level. Notably, interaction analyses suggested no synergistic or antagonistic statistical interaction. Furthermore, no evidence suggested that smoking status modified the association between the joint diet-sleep pattern and lung cancer risk.

In the stratified analysis by sex, the highest levels of the joint diet-sleep pattern were associated with a lower risk of lung cancer among females. Among males, inverse associations were observed for some intermediate-to-high levels, whereas the highest levels did not show similar associations. This may be explained by the limited number of events in males in levels 8 and 9 (20 or fewer events), which reduced statistical power. Overall, the findings were broadly suggestive of an inverse association in both sexes, although the pattern appeared more consistent in females and should be interpreted cautiously in males.

### Plausible biological mechanisms

The association we observed is biologically plausible, as both the Mediterranean diet and healthy sleep converge on shared pathways crucial for carcinogenesis.

The Mediterranean diet provides multiple polyphenols. As antioxidants, polyphenols can modulate the expression of specific microRNAs to suppress cancer cell-intrinsic survival pathways and inhibit cancer immune-evasion processes [4]. Moreover, the Mediterranean diet provides substantial dietary fiber. Short-chain fatty acids, which have anti-inflammatory and antioxidant effects, can be increasingly produced by gut bacteria through the fermentation of dietary fiber [28]. In addition, the low consumption of red meat results in a low level of heme iron, a pro-oxidant causing DNA damage [29]. In parallel, healthy sleep, particularly through the robust circadian secretion of melatonin, plays a vital role in alleviating oxidative stress and supporting immune function. These mechanisms include directly scavenging reactive oxygen and nitrogen species, indirectly boosting the activity of antioxidant enzymes, inhibiting pro-oxidant enzymes, and chelating transition metals that catalyze the production of free radicals [5, 30, 31]. We propose that these two factors jointly contribute to a systemic state characterized by reduced inflammation and enhanced antioxidant capacity. Importantly, such systemic effects may not influence all organs equally.

The pronounced inverse association observed specifically for lung cancer may be attributable to its unique physiological vulnerability. As the primary environmental interface, the lungs are perpetually exposed to high levels of exogenous and endogenous oxidative stress [32, 33]. This constant assault may make it particularly vulnerable to deficits in antioxidant and anti-inflammatory defenses. Therefore, the marginal benefit of a lifestyle that systemically bolsters these defenses is greatest for this organ, making the inverse association most detectable for lung cancer compared to other sites.

### Potential modifying role of siesta

The results indicated that although siesta was culturally linked to the Mediterranean diet, it did not appear to alter the association between Mediterranean diet adherence and lung cancer risk. Future studies with more detailed characterization of siesta are needed to confirm this finding.

### Strengths and limitations

The major strengths of our study include its prospective design, large sample size, long follow-up, and the comprehensive assessment of a wide range of covariates. The use of the Fine-Gray proportional subdistribution hazards model and multiple sensitivity analyses enhances the robustness of our findings.

However, several limitations must be acknowledged. First, as the Oxford WebQ did not capture all food components specified in the original MEDAS, the adapted MEDAS used in this study may not fully capture Mediterranean diet adherence as defined by the original MEDAS. Second, a substantial proportion of participants did not complete the Oxford WebQ, leading to their exclusion and a consequent reduction in sample size, which may affect the representativeness of the original population. Third, because few participants completed the dietary assessment at the initial dietary assessment, dietary data from both the initial and follow-up rounds were used, and temporal misalignment between exposure assessment and outcome follow-up cannot be fully excluded. Fourth, smoking was adjusted for using broad categories (never, previous, and current). This classification may not fully capture smoking intensity, cumulative exposure, duration, time since cessation, passive smoking, or other smoking-related behaviors. Thus, residual confounding by smoking cannot be excluded. Fifth, both diet and sleep were self-reported, which may have introduced measurement error and exposure misclassification. Sixth, the tertile-based classification of MEDAS and sleep scores was used to facilitate interpretation, but this approach may reduce information from the original scores. Finally, all participants were from the UK Biobank, which may limit the generalizability of our findings to other populations.

### Conclusion

In conclusion, the joint diet-sleep pattern was associated with a lower risk of lung cancer. These findings highlight the value of integrating lifestyle behaviors jointly in relation to lung cancer risk. We also found no evidence that the association between Mediterranean diet adherence and lung cancer risk was modified by siesta. However, causal effects cannot be inferred from this observational study. Future studies with repeated assessments of diet and sleep factors are needed to validate these findings and to further explore the underlying biological mechanisms.

## Supplementary Information

Below is the link to the electronic supplementary material.

Supplementary Material 1.

## Data Availability

The data underlying this study are available from the UK Biobank upon application and approval (https://www.ukbiobank.ac.uk/).
