# Supplementary material for: Mediterranean diet adherence, healthy sleep, and lung cancer risk: a prospective study of 192,034 UK Biobank participants
Source: Eur J Nutr. 2026 Aug 27;65(6):244. doi: 10.1007/s00394-026-04080-x (PMC13522091; doi:10.1007/s00394-026-04080-x)
Supplement: Supplementary file 1 [file 394_2026_4080_MOESM1_ESM.docx]

**Article title:** Mediterranean Diet Adherence, Healthy Sleep, and Lung Cancer Risk: A Prospective Study of 192,034 UK Biobank Participants

**Journal name:** European Journal of Nutrition

**Author names:** Yiming Chen, Enyu Tong, Xiaoman Jin, Yufeng Rao, Evan YW Yu, Abdulmohsen H Al-Zalabani, Maurice Zeegers, Anke Wesselius

**Affiliation and e-mail address of the corresponding author:** Anke Wesselius, Department of Epidemiology, Maastricht University, Maastricht 6229 ER, the Netherlands. E-mail: anke.wesselius@maastrichtuniversity.nl

**Supplementary Table S1** Selected variables with field IDs from the UK Biobank

| **Variable category** | **Variables with field IDs** |
| --- | --- |
| Dietary consumption variables | **Olive oil:** Type of fat/oil used for cooking (20090);  **Vegetables:** Carrot (104170), spinach (104300), broccoli (104140), cabbage/kale (104160), sprouts (104310), courgette (104200), cauliflower (104180), parsnip (104270), turnip/swede (104360), leek (104230), onion (104260), garlic (104220), mushroom (104250), sweet pepper (104290), side salad (104090), lettuce (104240), cucumber (104210), celery (104190), watercress (104370), fresh tomato (104340), tinned tomato (104350), sweetcorn (104320), beetroot (104130), avocado (104100), mixed vegetables (104060), vegetable pieces (104070), butternut squash (104150), other veg (104380), olives (102490), coleslaw (104080);  **Fruits:** Apple (104450), pear (104560), orange (104530), satsuma (104540), grapefruit (104490), banana (104460), grape (104500), melon (104520), peach/nectarine (104550), plum (104580), berry (104470), dried fruit (104430), stewed fruit (104410), mixed fruit (104440), prune (104420), cherry (104480), mango (104510), pineapple (104570), other fruit (104590);  **Red meat or red meat products:** Beef (103020), pork (103030), lamb (103040), bacon (103070), ham (103080), sausage (103010), liver (103090);  **Sweetened/carbonated drinks**: Fizzy drinks (100170), low calories drinks (100160), squash intake (100180);  **Wine:** Red wine (100590, 20096), rose wine (100630, 20097), white wine (100670, 20095);  **Legumes:** Peas (104280), green beans (104120), broad beans (104110), baked beans (104000), pulses (104010), tofu (103270);  **Fish/shellfish:** Battered fish (103180), breaded fish (103170), white fish (103190), oily fish (103160), shellfish (103220), other fish (103230), tinned tuna (103150), prawn (103200), lobster/crab (103210);  **Sweets/pastries:** Chocolate biscuit (102350), chocolate covered biscuit (102340), chocolate bar (102260), chocolate sweets (102310), chocolate raisins (102300), dark chocolate (102290), milk chocolate (102280), white chocolate (102270), sweet biscuits (102360), cakes (102190), cheesecake (102220), doughnut (102200), fruitcake (102180), Danish pastry (102060), sponge pudding (102210), milk based pudding (102140), other milk based pudding (102150), other dessert intake (102230), soya dessert intake (102170), sweets (102330), diet sweets (102320), other sweets (102380), ice cream (102120);  **Nuts:** Unsalted nuts (102440), salted nuts (102430), unsalted peanuts (102420), salted peanuts (102410);  **White meat:** Poultry (103060), breaded poultry (103050). |
| Sleep-related factors | Sleep duration (1160), insomnia (1200), snoring (1210), daytime dozing (1220), chronotype (1180), siesta (1190). |
| Cancer-related factors | Date of cancer diagnosis (40005), type of cancer (40006), reason for lost to follow-up (190), date of lost to follow-up (191), date of death (40000), date of attending assessment centre (53). |
| Covariates | Age at recruitment (21022), sex (31), coffee consumption (1498), smoking status (20116), alcohol status (20117), activity intensity (22032), household income (738), employment status (6142), Body Mass Index (21001). |

**Supplementary Table S2** Original version of the Mediterranean Diet Adherence Screener

| Questions | Scoring criteria for 0 point | Scoring criteria for 1 point |
| --- | --- | --- |
| 1. Do you use olive oil as your main culinary fat? | No | Yes |
| 2. How many tablespoons of olive oil do you consume per day? | <4/d | ≥4/d |
| 3. How many vegetable servings do you consume per day? | <2/d (and/or not including 1/d raw or salad) | ≥2/d (including ≥1/d raw or salad) |
| 4. How many servings of fresh fruit do you consume per day? | <3/d | ≥3/d |
| 5. How many servings of red meat, or red meat products do you consume per week? | ≥1/d | <1/d |
| 6. How many servings of butter, margarine, or cream do you consume per day? | ≥1/d | <1/d |
| 7. How many sweet/fizzy beverages/sodas do you consume per day? | ≥1/d | <1/d |
| 8. How many glasses/cups of wine do you consume per week? | <7/wk | ≥7/wk |
| 9. How many servings of legumes do you consume per week? | <3/wk | ≥3/wk |
| 10. How many servings of fish or shellfish do you consume per week? | <3/wk | ≥3/wk |
| 11. How many times per week do you consume industrial (not homemade) desserts/sweets/pastries? | ≥3/wk | <3/wk |
| 12. How many servings of nuts (including peanuts) do you consume per week? | <3/wk | ≥3/wk |
| 13. Do you preferentially consume chicken, turkey or rabbit meat, or a vegetarian protein source, instead of red meat or any derived products? | No | Yes |
| 14. How many times per week do you consume vegetables, pasta, rice or other dishes seasoned with sofrito? | <2/wk | ≥2/wk |

**Supplementary Table S3** Modified version of the Mediterranean Diet Adherence Screener

| **Questions** | **Scoring criteria for 0 point** | **Scoring criteria for 1 point** |
| --- | --- | --- |
| 1. Do you use olive oil? | Non-consumption | Consumption |
| 2. How many vegetable servings do you consume per day? | <2/d (and/or not including 1/d raw or salad) | ≥2/d (including ≥1/d raw or salad) |
| 3. How many servings of fresh fruit do you consume per day? | <3/d | ≥3/d |
| 4. How many servings of red meat, or red meat products do you consume per week? | ≥1/d | <1/d |
| 5. How many sweet/fizzy beverages/sodas do you consume per day? | ≥1/d | <1/d |
| 6. How many glasses/cups of wine do you consume per week? | <7/wk | ≥7/wk |
| 7. How many servings of legumes do you consume per week? | <3/wk | ≥3/wk |
| 8. How many servings of fish or shellfish do you consume per week? | <3/wk | ≥3/wk |
| 9. How many times per week do you consume industrial (not homemade) desserts/sweets/pastries? | ≥3/wk | <3/wk |
| 10. How many servings of nuts do you consume per week? | <3/wk | ≥3/wk |
| 11. Do you preferentially consume chicken, turkey or rabbit meat, or a vegetarian protein source, instead of red meat or any derived products? | No | Yes |

**Supplementary Table S4** The independent association between Mediterranean diet adherence and lung cancer risk

| **Variables** | **Model 1**^a^ | | **Model 2**^b^ | |
| --- | --- | --- | --- | --- |
|  | **SHR (95% CI)** | ***P*-value** | **SHR (95% CI)** | ***P*-value** |
| MEDAS score |  |  |  |  |
| Tertile 1 | 1 |  | 1 |  |
| Tertile 2 | **0.75 (0.65-0.87)** | **<0.001** | **0.82 (0.70-0.94)** | **0.006** |
| Tertile 3 | **0.70 (0.61-0.81)** | **<0.001** | **0.79 (0.69-0.92)** | **0.002** |
| Sex |  |  |  |  |
| Female | 1 |  |  |  |
| Male | 1.03 (0.92-1.16) | 0.560 | **0.88 (0.78-0.99)** | **0.037** |
| Age | **1.10 (1.10-1.11)** | **<0.001** | **1.11 (1.09-1.12)** | **<0.001** |
| Body Mass Index |  |  | 0.99 (0.97-1.00) | 0.056 |
| Coffee intake |  |  | **1.05 (1.03-1.08)** | **<0.001** |
| Alcohol status |  |  |  |  |
| Never |  |  | 1 |  |
| Previous |  |  | **1.95 (1.23-3.07)** | **0.004** |
| Current |  |  | 1.10 (0.73-1.64) | 0.650 |
| Smoking status |  |  |  |  |
| Never |  |  | 1 |  |
| Previous |  |  | **3.68 (3.14-4.31)** | **<0.001** |
| Current |  |  | **12.15 (10.24-14.41)** | **<0.001** |
| Activity intensity |  |  |  |  |
| Low |  |  | 1 |  |
| Moderate |  |  | **0.80 (0.68-0.93)** | **0.004** |
| High |  |  | **0.76 (0.65-0.89)** | **0.001** |
| Household income |  |  |  |  |
| Less than 18,000 |  |  | 1 |  |
| 18,000 to 30,999 |  |  | **0.85 (0.73-0.99)** | **0.036** |
| 31,000 to 51,999 |  |  | **0.68 (0.58-0.81)** | **<0.001** |
| 52,000 to 100,000 |  |  | **0.63 (0.52-0.77)** | **<0.001** |
| Greater than 100,000 |  |  | **0.73 (0.53-0.99)** | **0.045** |
| Employment status |  |  |  |  |
| Employed |  |  | 1 |  |
| Retired |  |  | 0.92 (0.78-1.08) | 0.320 |
| Other status |  |  | 1.04 (0.88-1.24) | 0.630 |

MEDAS: the Mediterranean Diet Adherence Screener; SHR: subdistribution hazard ratio; CI: confidence interval.

^a^ adjusted for age and sex.

^b^ further adjusted for all covariates.

**Supplementary Table S5** The independent association between sleep and lung cancer risk

| **Variables** | **Model 1**^a^ | | **Model 2**^b^ | |
| --- | --- | --- | --- | --- |
|  | **SHR (95% CI)** | ***P*-value** | **SHR (95% CI)** | ***P*-value** |
| Sleep score |  |  |  |  |
| Tertile 1 | 1 |  | 1 |  |
| Tertile 2 | **0.88 (0.77-0.99)** | **0.037** | 0.98 (0.86-1.11) | 0.710 |
| Tertile 3 | **0.60 (0.51-0.72)** | **<0.001** | **0.74 (0.62-0.88)** | **0.001** |
| Sex |  |  |  |  |
| Female | 1 |  |  |  |
| Male | 1.05 (0.93-1.18) | 0.410 | 0.90 (0.80-1.01) | 0.072 |
| Age | **1.10 (1.09-1.11)** | **<0.001** | **1.10 (1.09-1.12)** | **<0.001** |
| Body Mass Index |  |  | 0.99 (0.97-1.00) | 0.059 |
| Coffee intake |  |  | **1.05 (1.03-1.08)** | **<0.001** |
| Alcohol status |  |  |  |  |
| Never |  |  | 1 |  |
| Previous |  |  | **1.94 (1.23-3.06)** | **0.004** |
| Current |  |  | 1.07 (0.71-1.59) | 0.760 |
| Smoking status |  |  |  |  |
| Never |  |  | 1 |  |
| Previous |  |  | **3.62 (3.09-4.23)** | **<0.001** |
| Current |  |  | **12.04 (10.15-14.29)** | **<0.001** |
| Activity intensity |  |  |  |  |
| Low |  |  | 1 |  |
| Moderate |  |  | **0.80 (0.68-0.93)** | **0.004** |
| High |  |  | **0.76 (0.65-0.89)** | **0.001** |
| Household income |  |  |  |  |
| Less than 18,000 |  |  | 1 |  |
| 18,000 to 30,999 |  |  | **0.85 (0.73-0.98)** | **0.028** |
| 31,000 to 51,999 |  |  | **0.67 (0.57-0.79)** | **<0.001** |
| 52,000 to 100,000 |  |  | **0.61 (0.50-0.75)** | **<0.001** |
| Greater than 100,000 |  |  | **0.70 (0.51-0.95)** | **0.023** |
| Employment status |  |  |  |  |
| Employed |  |  | 1 |  |
| Retired |  |  | 0.92 (0.79-1.08) | 0.320 |
| Other status |  |  | 1.04 (0.87-1.24) | 0.660 |

SHR: subdistribution hazard ratio; CI: confidence interval.

^a^ adjusted for age and sex.

^b^ further adjusted for all covariates.

**Supplementary Table S6** The associations between the joint diet-sleep pattern and risk of other cancers

| **Cancer type** | **Joint diet-sleep pattern** | **Model 1**^a^ | | **Model 2**^b^ | |
| --- | --- | --- | --- | --- | --- |
|  |  | **SHR (95% CI)** | ***P*-value** | **SHR (95% CI)** | ***P*-value** |
| Breast cancer | Level 1 | 1 |  | 1 |  |
|  | Level 2 | 0.97 (0.86-1.09) | 0.610 | 0.98 (0.87-1.11) | 0.750 |
|  | Level 3 | 1.00 (0.89-1.12) | 0.990 | 1.02 (0.91-1.14) | 0.770 |
|  | Level 4 | 0.91 (0.82-1.01) | 0.065 | 0.93 (0.84-1.03) | 0.170 |
|  | Level 5 | 0.92 (0.82-1.03) | 0.170 | 0.95 (0.84-1.07) | 0.380 |
|  | Level 6 | **0.88 (0.79-0.98)** | **0.021** | 0.91 (0.81-1.02) | 0.093 |
|  | Level 7 | 0.93 (0.82-1.04) | 0.190 | 0.96 (0.86-1.08) | 0.530 |
|  | Level 8 | 0.88 (0.77-1.01) | 0.061 | 0.92 (0.80-1.05) | 0.230 |
|  | Level 9 | **0.88 (0.77-1.00)** | **0.046** | 0.92 (0.81-1.05) | 0.230 |
| Colorectal cancer | Level 1 | 1 |  | 1 |  |
|  | Level 2 | 1.07 (0.91-1.25) | 0.420 | 1.08 (0.92-1.26) | 0.330 |
|  | Level 3 | 0.94 (0.80-1.10) | 0.430 | 0.96 (0.81-1.13) | 0.600 |
|  | Level 4 | 0.92 (0.81-1.05) | 0.210 | 0.95 (0.83-1.08) | 0.440 |
|  | Level 5 | 0.89 (0.76-1.04) | 0.140 | 0.92 (0.79-1.08) | 0.330 |
|  | Level 6 | 0.88 (0.75-1.03) | 0.110 | 0.92 (0.79-1.08) | 0.310 |
|  | Level 7 | 0.87 (0.74-1.03) | 0.096 | 0.91 (0.77-1.08) | 0.290 |
|  | Level 8 | 0.91 (0.74-1.11) | 0.330 | 0.96 (0.79-1.18) | 0.700 |
|  | Level 9 | 0.88 (0.73-1.07) | 0.200 | 0.95 (0.78-1.15) | 0.590 |
| Prostate cancer | Level 1 | 1 |  | 1 |  |
|  | Level 2 | 1.02 (0.92-1.13) | 0.710 | 0.99 (0.90-1.10) | 0.920 |
|  | Level 3 | 1.05 (0.93-1.19) | 0.390 | 1.01 (0.89-1.13) | 0.930 |
|  | Level 4 | 0.92 (0.83-1.03) | 0.150 | 0.91 (0.81-1.01) | 0.083 |
|  | Level 5 | 1.01 (0.91-1.12) | 0.860 | 0.97 (0.88-1.07) | 0.530 |
|  | Level 6 | 1.02 (0.90-1.14) | 0.790 | 0.96 (0.85-1.08) | 0.450 |
|  | Level 7 | 0.94 (0.81-1.08) | 0.370 | 0.91 (0.78-1.05) | 0.170 |
|  | Level 8 | 0.98 (0.87-1.11) | 0.760 | 0.93 (0.82-1.05) | 0.260 |
|  | Level 9 | 0.99 (0.85-1.16) | 0.930 | 0.92 (0.79-1.08) | 0.310 |
| Gastric cancer | Level 1 | 1 |  | 1 |  |
|  | Level 2 | 0.72 (0.43-1.21) | 0.220 | 0.77 (0.46-1.30) | 0.330 |
|  | Level 3 | 1.01 (0.63-1.61) | 0.980 | 1.13 (0.71-1.81) | 0.600 |
|  | Level 4 | 1.01 (0.70-1.47) | 0.950 | 1.09 (0.75-1.58) | 0.670 |
|  | Level 5 | 0.94 (0.59-1.49) | 0.780 | 1.07 (0.67-1.70) | 0.780 |
|  | Level 6 | 0.60 (0.35-1.02) | 0.059 | 0.71 (0.42-1.22) | 0.220 |
|  | Level 7 | 0.84 (0.51-1.37) | 0.480 | 0.95 (0.57-1.57) | 0.830 |
|  | Level 8 | 0.61 (0.30-1.22) | 0.160 | 0.72 (0.35-1.47) | 0.370 |
|  | Level 9 | 0.49 (0.23-1.04) | 0.065 | 0.62 (0.29-1.32) | 0.220 |
| Liver cancer | Level 1 | 1 |  | 1 |  |
|  | Level 2 | 0.86 (0.52-1.42) | 0.560 | 0.92 (0.56-1.53) | 0.760 |
|  | Level 3 | 1.02 (0.64-1.63) | 0.940 | 1.14 (0.71-1.83) | 0.590 |
|  | Level 4 | 0.88 (0.59-1.30) | 0.510 | 0.96 (0.64-1.44) | 0.850 |
|  | Level 5 | 0.82 (0.50-1.34) | 0.430 | 0.96 (0.58-1.58) | 0.860 |
|  | Level 6 | 0.80 (0.49-1.29) | 0.360 | 0.97 (0.59-1.58) | 0.890 |
|  | Level 7 | 0.83 (0.50-1.37) | 0.460 | 0.97 (0.58-1.61) | 0.900 |
|  | Level 8 | 1.37 (0.81-2.29) | 0.240 | 1.68 (1.00-2.82) | 0.050 |
|  | Level 9 | 0.61 (0.31-1.19) | 0.150 | 0.79 (0.40-1.56) | 0.490 |
| Thyroid cancer | Level 1 | 1 |  | 1 |  |
|  | Level 2 | **0.43 (0.20-0.91)** | **0.028** | **0.44 (****0.21-****0.94)** | **0.035** |
|  | Level 3 | 0.60 (0.31-1.16) | 0.130 | 0.65 (0.34-1.26) | 0.200 |
|  | Level 4 | 1.09 (0.70-1.70) | 0.700 | 1.14 (0.74-1.76) | 0.560 |
|  | Level 5 | 0.60 (0.32-1.13) | 0.110 | 0.65 (0.35-1.23) | 0.180 |
|  | Level 6 | 0.82 (0.47-1.42) | 0.480 | 0.91 (0.53-1.58) | 0.750 |
|  | Level 7 | 0.71 (0.39-1.30) | 0.270 | 0.75 (0.41-1.38) | 0.360 |
|  | Level 8 | 0.62 (0.29-1.33) | 0.220 | 0.68 (0.32-1.48) | 0.330 |
|  | Level 9 | **0.40 (0.17-0.95)** | **0.037** | 0.45 (0.19-1.09) | 0.077 |
| Cervical cancer | Level 1 | 1 |  | 1 |  |
|  | Level 2 | 1.42 (0.81-2.49) | 0.220 | 1.48 (0.85-2.60) | 0.170 |
|  | Level 3 | 0.96 (0.52-1.78) | 0.900 | 1.00 (0.54-1.86) | 0.999 |
|  | Level 4 | 1.29 (0.81-2.03) | 0.280 | 1.36 (0.86-2.15) | 0.190 |
|  | Level 5 | 1.26 (0.74-2.14) | 0.400 | 1.35 (0.79-2.30) | 0.270 |
|  | Level 6 | 1.12 (0.65-1.91) | 0.680 | 1.22 (0.71-2.10) | 0.470 |
|  | Level 7 | 0.90 (0.51-1.59) | 0.720 | 0.98 (0.56-1.74) | 0.950 |
|  | Level 8 | 0.91 (0.47-1.77) | 0.780 | 1.03 (0.53-2.00) | 0.930 |
|  | Level 9 | 0.87 (0.46-1.67) | 0.680 | 0.99 (0.51-1.94) | 0.980 |
| Bladder cancer | Level 1 | 1 |  | 1 |  |
|  | Level 2 | 0.93 (0.72-1.21) | 0.600 | 0.96 (0.74-1.24) | 0.740 |
|  | Level 3 | 0.95 (0.73-1.23) | 0.700 | 0.98 (0.76-1.28) | 0.900 |
|  | Level 4 | 0.82 (0.66-1.01) | 0.063 | 0.87 (0.70-1.07) | 0.180 |
|  | Level 5 | 0.98 (0.77-1.25) | 0.870 | 1.05 (0.82-1.35) | 0.700 |
|  | Level 6 | 0.82 (0.63-1.06) | 0.130 | 0.88 (0.68-1.15) | 0.340 |
|  | Level 7 | 0.98 (0.76-1.26) | 0.850 | 1.08 (0.83-1.39) | 0.570 |
|  | Level 8 | 1.08 (0.80-1.46) | 0.620 | 1.20 (0.88-1.63) | 0.250 |
|  | Level 9 | 0.72 (0.51-1.02) | 0.065 | 0.81 (0.57-1.16) | 0.250 |
| Non-Hodgkin lymphoma | Level 1 | 1 |  | 1 |  |
|  | Level 2 | 0.89 (0.67-1.18) | 0.430 | 0.90 (0.68-1.19) | 0.470 |
|  | Level 3 | 0.98 (0.75-1.28) | 0.870 | 1.00 (0.76-1.31) | 0.980 |
|  | Level 4 | 1.04 (0.84-1.30) | 0.700 | 1.05 (0.85-1.31) | 0.640 |
|  | Level 5 | 1.04 (0.80-1.34) | 0.790 | 1.06 (0.82-1.37) | 0.670 |
|  | Level 6 | 0.90 (0.69-1.17) | 0.440 | 0.92 (0.71-1.21) | 0.560 |
|  | Level 7 | 1.01 (0.77-1.32) | 0.950 | 1.03 (0.78-1.34) | 0.850 |
|  | Level 8 | 0.87 (0.61-1.22) | 0.420 | 0.89 (0.63-1.26) | 0.510 |
|  | Level 9 | 0.87 (0.63-1.21) | 0.420 | 0.90 (0.65-1.26) | 0.550 |

SHR: subdistribution hazard ratio; CI: confidence interval.

^a^ adjusted for age and sex.

^b^ further adjusted for all covariates.
